# Breastfeeding, pregnancy, medicines, neurodevelopment, and population databases: the information desert

**DOI:** 10.1186/s13006-022-00494-5

**Published:** 2022-08-02

**Authors:** Sue Jordan, Rebecca Bromley, Christine Damase-Michel, Joanne Given, Sophia Komninou, Maria Loane, Naomi Marfell, Helen Dolk

**Affiliations:** 1 Faculty of Medicine, Health and Life Science, Swansea University, Swansea, Wales UK; 2 Division of Neuroscience and Experimental Psychology, School of Biological Sciences, Faculty of Biology, Medicine and Health, University of Manchester, Manchester, UK; 3 Royal Manchester Children’s Hospital, Central Manchester University Hospitals NHS Foundation Trust, Manchester Academic Health Science Centre, Manchester, UK; 4 Faculté de Médecine, Center for Epidemiology and Research in POPulation Health (CERPOP), Université Toulouse III, CHU Toulouse INSERM, Pharmacologie Médicale, Toulouse, France; 5 Faculty Life & Health Sciences, University of Ulster, Co Antrim, Newtownabbey, N Ireland UK

**Keywords:** Breastfeeding, Pharmacoepidemiology, Pharmacovigilance, Pregnancy, Child development, Adverse drug reactions

## Abstract

**Background:**

The pharmacoepidemiology of the long-term benefits and harms of medicines in pregnancy and breastfeeding has received little attention. The impact of maternal medicines on children is increasingly recognised as a source of avoidable harm. The focus of attention has expanded from congenital anomalies to include less visible, but equally important, outcomes, including cognition, neurodevelopmental disorders, educational performance, and childhood ill-health. Breastfeeding, whether as a source of medicine exposure, a mitigator of adverse effects or as an outcome, has been all but ignored in pharmacoepidemiology and pharmacovigilance: a significant ‘blind spot’.

**Whole-population data on breastfeeding: why we need them:**

Optimal child development and maternal health necessitate breastfeeding, yet little information exists to guide families regarding the safety of medicine use during lactation. Breastfeeding initiation or success may be altered by medicine use, and breastfeeding may obscure the true relationship between medicine exposure during pregnancy and developmental outcomes. Absent or poorly standardised recording of breastfeeding in most population databases hampers analysis and understanding of the complex relationships between medicine, pregnancy, breastfeeding and infant and maternal health. The purpose of this paper is to present the arguments for breastfeeding to be included alongside medicine use and neurodevelopmental outcomes in whole-population database investigations of the harms and benefits of medicines during pregnancy, the puerperium and postnatal period. We review: 1) the current situation, 2) how these complexities might be accommodated in pharmacoepidemiological models, using antidepressants and antiepileptics as examples; 3) the challenges in obtaining comprehensive data**.**

**Conclusions:**

The scarcity of whole-population data and the complexities of the inter-relationships between breastfeeding, medicines, co-exposures and infant outcomes are significant barriers to full characterisation of the benefits and harms of medicines during pregnancy and breastfeeding. This makes it difficult to answer the questions: ‘is it safe to breastfeed whilst taking this medicine’, and ‘will this medicine interfere with breastfeeding and/ or infants’ development’?

## Background

### Pharmacoepidemiology, pharmacovigilance and the reproductive years

After a medicinal product has been marketed, patient safety depends on accurate population surveillance, pharmacovigilance^glossary^—detecting, assessing, and preventing adverse effects, and pharmacoepidemiology^glossary^ – describing the use and effects of drugs in large numbers of people (Table 1 Glossary has definitions). Pregnant and breastfeeding individuals and their infants should not be excluded from the protection afforded by pharmacovigilance [1]. Neither continuation nor discontinuation of medicines is without risk, but harm can be minimised by effective pharmacovigilance. This depends on comprehensive characterisation of drug-related benefits and harms, and any imbalance. For people of childbearing age, this should include information across the full reproductive life cycle: fertility rates; pregnancy loss; terminations, for all reasons; congenital anomalies; preterm birth; growth centiles; complications of pregnancy; complications of childbirth and the puerperium (e.g. haemorrhage); neonatal complications (pulmonary hypertension, hypoglycaemia, discontinuation syndromes); breastfeeding rates at different ages; infant and childhood outcomes, including cognitive functioning, neurodevelopmental disorders, education performance, long-term conditions, survival and reproductive success [2].

**Table 1:** Glossary: definitions of terms in this paper

	**Definition(s)**	**Reference**
Adverse drug reaction, ADR	Noxious and unintended responses to pharmacotherapy (or medicines) A response to a medicinal product which is noxious and unintended. This includes adverse reactions which arise from: • The use of a medicinal product within the terms of the marketing authorisation • The use outside the terms of the marketing authorisation, including overdose, off-label use, misuse, abuse and errors • Occupational exposure European Medicines Agency (2017)p.6 A transgenerational ADR is across generations, affecting the child, not the parent taking the medicine.	[3] European Medicines Agency (EMA) Guideline on good pharmacovigilance (GVP). Module VI – Collection, management and submission of reports of suspected adverse reactions to medicinal products (Rev 2). 2017. Available: http://www.ema.europa.eu/docs/en_GB/document_library/Regulatory_and_procedural_guideline/2017/08/WC500232767.pdf ‘Trans’ is the prefix used to denote ‘across / from one another’ (OED trans-, prefix: Oxford English Dictionary (oed.com))
Bias	‘Any process at any stage of inference which tends to produce results or conclusions that differ systematically from the truth’. (Adapted from Murphy. The Logic of Medicine. Baltimore: John Hopkins University Press. 1976)	[4] Sackett DL: Bias in analytic research. J Chronic Dis 1979, 32(1–2): 51–63
Collider bias	The distortion that occurs when two variables independently cause a third variable (the collider), and the analysis is conditioned on the third variable (the collider). The condition may be as a restriction (or condition) of study entry or as a covariate in a regression model. This is selection bias based on 2 or more variables.	[5] Munafò MR, Tilling K, Taylor AE, Evans DM, Davey Smith G: Collider scope: when selection bias can substantially influence observed associations. Int J Epidemiol 2018, 47(1): 226–235. P.227 Cole SR, Platt RW, Schisterman EF, Chu H, Westreich D, Richardson D, Poole C: Illustrating bias due to conditioning on a collider. International Journal of Epidemiology 2010, 39(2): 417–420, p.419
Confounding variable	A variable (measured or not) other than the predictor variables of interest that potentially affects the outcome variable. P.783	[6] Field A. Discovering statistics using spss. London: Sage; 2013 4^th^ edition
Confounder	A factor associated with both the exposure (predictor) and the outcome, and not part of the causal pathway from exposure to outcome. P.195 Other definitions exist, some specify that the confounder is present before the exposure [7] (VanderWeele TJ, Shpitser I. On the definition of a confounder. Ann Stat. 2013, 41(1):196–220. 10.1214/12-aos1058) The Oxford English Dictionary definition is: ‘One who causes confusion or disorder, who confuses distinctions’.	[8] Kahlert J, Gribsholt SB, Gammelager H, Dekkers OM, Luta G: Control of confounding in the analysis phase – an overview for clinicians. Clin Epidemiol 2017, 9:195–204. 10.2147/CLEP.S129886
Covariate	Any variable that is measurable and considered to have a statistical relationship with the outcome variable is a potential covariate. A covariate is a possible predictive or explanatory variable of the outcome. P.2	[9] Salkind, NJ: Encyclopedia of Research Design (Vols. 1–0). Thousand Oaks, CA: SAGE Publications, Inc; 2010 https://doi.org/10.4135/9781412961288
Deprivation score	Deprivation scores, ranks and quintiles are based on small geographical areas of residence. The UK’s Townsend measure of material deprivation, one of the first of these to have been created, is calculated from rates of unemployment, vehicle ownership, home ownership, and overcrowding (Townsend, 1988). Such scores are unavailable in countries without area-based codes, increasing reliance on other measures, such as income and maternal time in education.	[10] Townsend P, Phillimore P, Beattie A: Health and Deprivation. London: Routledge; 1988
Determinant	A determining factor or agent; a ruling antecedent, a conditioning element; a defining word or element.	[11] Oxford English Dictionary (OED) Online
Marginal Structual Models	A class of causal models for the estimation, from observational data, of the causal effect of a time-dependent exposure (treatment) in the presence of time-dependent covariates that may be simultaneously confounders and intermediate variables (e.g. breastfeeding).	[12] Robins JM, Hernán MA, Brumback B. Marginal structural models and causal inference in epidemiology. Epidemiology. 2000 Sep;11(5):550–60. https://doi.org/10.1097/00001648-200009000-00011. PMID: 10,955,408
Mediator variable	An entity or process that intervenes between input and output. A variable functions as a mediator to the extent that it accounts for the relation between the predictor and the outcome. Whereas moderator variables specify when certain effects will hold, mediators speak to how or why such effects occur. This can be illustrated as a causal chain. A variable functions as a mediator when: a) variations in levels of the predictor variable significantly account for variations in the presumed mediator, b) variations in the mediator significantly account for variations in the outcome variable, and c) when these are both controlled, a previously significant relation between the predictor and outcome variables is no longer significant. P.1176	[13] Baron RM, Kenny DA: The moderator–mediator variable distinction in social psychological research: Conceptual, strategic, and statistical considerations. Journal of Personality and Social Psychology 1986, 51(6):1173–1182. 10.1037/0022-3514.51.6.1173
Moderator variable / effect modifier	A qualitative (e.g., sex, race, class) or quantitative variable that affects the direction and/or strength of the relation between a predictor variable and an outcome variable. Specifically, within a correlational analysis framework, a moderator is a third variable that affects the zero-order correlation between two other variables. P.1174	[13] Baron RM, Kenny DA: The moderator–mediator variable distinction in social psychological research: Conceptual, strategic, and statistical considerations. Journal of Personality and Social Psychology 1986, 51(6):1173–1182. 10.1037/0022-3514.51.6.1173
Multi-level modelling	A strategy to account for clustering of participants e.g. by primary care provider or school. Where participants are clustered, exposures and outcomes may not be independent. This hierarchical analysis is used in educational effectiveness studies to explore the influence of individual schools and classes.	[14] Miles J. & Shelvin M. 2001 Applying Regression and Correlation. Sage, London. P.192
Parameter	A term with extended and technical uses in many disciplines, including statistics, music, geometry. In general usage: any distinguishing or defining characteristic or feature, esp. one that may be measured or quantified; an element or aspect of something; (more widely) a boundary or limit.	[11] Oxford English Dictionary (OED) Online https://www.oed.com/view/Entry/137519?redirectedFrom=parameter#eid
Pharmaco-epidemiology	Pharmacoepidemiology is the study of the use and effects of drugs in large numbers of people (WHO 2002 p.42); by applying epidemiological methods to pharmacology questions it bridges two disciplines.	[15] World Health Organization (2002). The importance of pharmacovigilance, safety monitoring of medicinal products. Geneva. https://apps.who.int/iris/bitstream/handle/10665/42493/a75646.pdf?sequence=1&isAllowed=y
Pharmaco-vigilance	Pharmacovigilance, a branch of pharmacoepidemiology, is the science and activities relating to the detection, assessment, understanding and prevention of adverse effects or any other possible drug-related problems (WHO 2002 p.7).	[15] World Health Organization (2002). The importance of pharmacovigilance, safety monitoring of medicinal products. Geneva. https://apps.who.int/iris/bitstream/handle/10665/42493/a75646.pdf?sequence=1&isAllowed=y
Regression / multiple regression	An equation (or model) where the outcome is predicted by a combination of ≥ 2 predictor (or exposure or input) variables. The model assigns a regression coefficient to each predictor variable, whose statistical significance can then be calculated.	[6] Field A. Discovering statistics using spss. London: Sage; 2013 4^th^ edition
Risk factor	A risk factor a) precedes the outcome, and b) when used it divides a population into high risk and low risk subgroups. Risk factors may be population specific. They may be fixed markers, variable markers or causal risk factors.	[16] Offord DR, Kraemer HC Risk factors and prevention Evidence-Based Mental Health 2000;3:70–71
Selection bias	The introduction of error due to systematic differences in the characteristics between those selected and those not selected for a given study.	[17] PubMed MeSH database (1990) Selection bias: http://www.ncbi.nlm.nih.gov/mesh?term=selection%20bias. Accessed 14 December 2012
Socio-economic status, SES	Social class, social stratification, social or SES or position, are often used interchangeably. Socioeconomic status (SES) is a combination of economic and social factors (income, education, housing tenure, occupation) that influence the positions individuals or groups hold within the structure of a society (Krieger, 1997). It encompasses concepts with different theoretical, historical and disciplinary origins (Galobardes, 2006). SES is a relative, not absolute, measure. The most disadvantaged of some countries may be better situated than the most advantaged of others	[18] Krieger N, Williams DR, Moss NE: Measuring social class in US public health research: concepts, methodologies, and guidelines. Annu Rev Public Health 1997, 18:341–378 [19] Galobardes B, Shaw M, Lawlor DA, Lynch JW, Davey Smith G: Indicators of socioeconomic position (part 1). J Epidemiol Community Health 2006, 60(1):7–12. 10.1136/jech.2004.023531
Structural equation modelling	A general analytic technique for testing complex hypotheses that cannot be adequately described in regression models.	[14] Miles J. & Shelvin M. 2001 Applying Regression and Correlation. Sage, London. P.199
Variable	Anything that varies within a set of data. P.17	[20] Altman DG: Practical Statistics for Medical Research. London: Chapman & Hall; 1991
Variance	In statistics, a measure of dispersion, the square of the standard deviation or the sum of the distances between the observations and the mean divided by (n-1). P.34	[20] Altman DG: Practical Statistics for Medical Research. London: Chapman & Hall; 1991
Volunteer bias	Any process which tends to produce results or conclusions that differ systematically from the truth, arising where volunteers from a specified sample may exhibit exposures or outcomes which differ from those of non-volunteers. P.2	[21] Jordan S, Watkins A, Storey M, Allen SJ, Brooks CJ, Garaiova I, Heaven ML, Jones R, Plummer SF, Russell IT, Thornton CA, Morgan G. (2013) Volunteer Bias in Recruitment, Retention, and Blood Sample Donation in a Randomised Controlled Trial Involving Mothers and Their Children at Six Months and Two Years: A Longitudinal Analysis. PLoS ONE 8(7): e67912. 10.1371/journal.pone.0067912

This paper aims to present the arguments for breastfeeding to be included alongside medicines use and neurodevelopmental outcomes in population databases and studies investigating the benefits and harms of medicines during pregnancy, the puerperium and postnatal period. We review:

- 1) the current situation.
- 2) how breastfeeding might be accommodated in pharmacoepidemiological models exploring the impact of medicines on breastfeeding as an outcome and on infants exposed to medicines *in utero* and via breastmilk.
- 3) the challenges in obtaining comprehensive data.

#### Breastfeeding and medicines: locating the data and why we need them

To research the impact of medicine exposure during and after pregnancy on infant development, we shall need population databases linking data on medicine exposure plus breastfeeding plus infant development. Across Europe, few population databases hold data on all three together, and there is little uniformity in outcomes, definitions, methods and timing of assessments, as illustrated in Table 2.

**Table 2:** European Population-based data sources with data on breastfeeding plus medicines use during pregnancy plus neurodevelopment

Country	Data sources (breastfeeding data sources italicised)	Neurodevelopmental measurement available	Breastfeeding information categories as they appear in the data source	Pregnancies per year (1,000 s)	Birth years with breastfeeding plus neurodevelopment data
**Finland**	Care Register for Health Care, Primary Health Care, Drugs and Pregnancy Database, *Finnish Medical Birth Register*, CA registry	ICD codes recorded in outpatient or GP care	Assessed and recorded by midwives at discharge or 7 days *postpartum* Categories: exclusive breastfeeding, partial breastfeeding, ‘artificial milk’ only	50	2017-
France (Haute-Garonne)	EFEMERIS* *database (pregnant women and their children)*	Certificates completed at 9 and 24 months by a general practitioner or a paediatrician – include 14 items designed to detect children at risk of psychomotor development abnormalities	Self-report, recorded on health certificates completed during mandatory medical examinations at 8 days, 9 months and 24 months	10	mid 2004-
	POMME *databases (breastfeeding data to 24 months)*	As above plus Medicines and health care reimbursements	Categories: ‘any’ breastfeeding (Yes/No), duration of breastfeeding (in weeks), and duration of exclusive breastfeeding (weeks) (Both databases)	18.5	Follow up of birth years: mid 2010 to mid 2011 + mid 2015 to mid 2016
**Italy – Tuscany**	Mental health services, *birth registry*, medicines dispensed in community pharmacies, and hospital pharmacies for outpatient use	Outpatient and mental health service ICD codes	Hospital records documenting how the new-born was fed during the hospital stay Categories: Only breast milk, breast milk with the addition of water or liquids other than milk, breast milk and infant formula, infant formula only	30	2010-
UK- Scotland	*Child Health Systems, Programme – Pre-School, Child Health Systems Programme – School,* Support Needs System, Maternity hospital discharge records (including delivery records), Prescribing Information System	Children registered on the Support Needs System, Child health developmental examinations	Health visitors’ records of self-report at 10 days, 6 weeks and 13 months. Categories: breast milk only, fed formula milk only, or fed both breast and formula milk	53	2013-
**UK – Wales**	In-patient and out-patient records, Primary Care GP data†, *National Community Child Health Database,* National Pupil Database Wales, congenital anomaly registry	ICD/Read codes, child health developmental examinations, special education needs, and educational attainment from 7 to 16 years	Health visitors’ records of self-report: at birth and 6–8 weeks at 6 and 12 months Categories: ‘any’ breastfeeding (yes/no)	33	2005- 2015-

Information was collated in January 2021. Data sources were identified by contacting representatives of all countries in Europe and searching the literature to compile the Fair Data Catalogue for the Conception project, as described:. https://www.imi-conception.eu/wp-content/uploads/2019/09/ConcePTION_D1.1_spreadsheet-containing-all-additional-data-sources-for-the-ConcePTION-Data-Source-Catalogue.pdf To identify data sources containing all three variables, the breastfeeding and neurodevelopmental data source lists were cross-referenced and data were discussed with the data access providers

*ICD* International classification of disease, as issued by the World Health Organisation (WHO)

We use ‘neurodevelopment’ as an umbrella term for cognitive, social, motor and behavioural development. How these data can be usefully combined and standardised is being investigated

Exclusive breastfeeding is as defined by the WHO (2008): Infant receives only breast milk from his/her mother or a wet nurse, or expressed breast milk via tube, cup or syringe, and no other liquids or solids with the exception of drops or syrups consisting of vitamins, mineral supplements or medicine. (*WHO 2008 Indicators for Assessing Infant and Young Child Feeding Practices – Part I: Definitions. Conclusions of a Consensus Meeting Held 6–8 November 2007 in Washington D.C.* https://apps.who.int/iris/bitstream/handle/10665/43895/9789241596664_eng.pdf;jsessionid=DB32B0C8C42A0F61174ECAF42D8FC8FD?sequence=1)

Where breastfeeding is self-reported at certain time-points, the duration of ‘breastmilk only’ or ‘any breastfeeding’ is taken as ‘from birth’. We acknowledge this may introduce imprecision

^*^EFEMERIS covers the 80% of the population covered by the state-controlled French Health Insurance [22]

^†^In Wales, ~ 80% primary care providers voluntarily supply medicines data to the databank. Any selection bias is due to healthcare providers, not subjects. All pregnancies identified can be followed for life, unless the individual leaves the country

Papers relating medicines use to breastfeeding are available for France [23] and Wales [24, 25]

It took 30 years for the dose–response associations between *in utero* exposure to valproic acid derivatives and altered neurodevelopment trajectories [26], and congenital anomalies [27] to be incorporated into regulatory measures to curtail prescribing during pregnancy [28]. Valproate prescribing to women of childbearing age is declining [29], but, in England, it was prescribed to 247 pregnant women between April 2018 and September 2021 [30]. Subsequently, pharmacoepidemiologists have considered the effects of exposure to other medicines during pregnancy, particularly, antidepressants [31], and opioids [32]. However, although suboptimal breastfeeding is one of the main threats to global health [33], it appears to be a ‘blind spot’ in healthcare databases and pharmacovigilance.

It is rarely possible to obtain the population-wide picture of short- and long-term transgenerational outcomes from clinical trials, case series, spontaneous reports, and cohort studies, due to (largely unavoidable) selection bias^glossary^ [21, 34]. Prospective observational birth cohort studies collect information on infant feeding, but they represent a self-selected sample of the population. For example, cohorts in Norway and Denmark recruited 41% and ~ 30% of the eligible populations [35, 36], whilst other cohorts lack linked prescription [37] or child development data [38]. Pharmacokinetic studies usually involve small numbers of participants, and, like animal studies, may not predict developmental or clinical outcomes [39]. Manufacturers’ medicine-related pregnancy registries capture insufficient data on pregnancy, infant follow-up, and breastfeeding: median (interquartile range) enrolment is reported as 36 (5–258) pregnancies and 12 (2–119) infants [40]. Patient safety researchers are, therefore, examining population databases; however, data quality and validity are not always completely evaluated [41, 42], and most have no data on breastfeeding and neurodevelopment [43, 44].

##### Breastfeeding

Breastfeeding requires an optimal biopsychosocial *milieu* [45]. Socioeconomic status (SES) ^glossary^, cultural norms, availability of infant formula, and maternal intention are important, but the impact of prescribed medicines on breastfeeding should not be overlooked [46]. Exposure to some prescription medicines in pregnancy, labour and *postpartum*, may reduce breastfeeding initiation or continuation [24, 46, 47]. The complex physiology of lactation is vulnerable to disruption, particularly by medicines that affect serotoninergic pathways (including antidepressants) [48, 49], antagonise prolactin (amphetamines, oestrogens, ergotamine derivatives, aripiprazole, promethazine, possibly diuretics, injected corticosteroids), or reduce oxytocin release (alcohol, opioids, possibly sympathomimetics, anticholinergics, antidepressants) [50–53]. However, we do not know all the reasons underlying the lower breastfeeding rates amongst those using prescription medicines. People may be reluctant to breastfeed if the impact of the medicine on the infant is unknown [54]: doubts, hesitancies, and anxieties may compound physiological difficulties.

##### Neurodevelopment

Medicine exposure through breastfeeding is an important consideration in analyses of neurodevelopmental outcomes (neurodevelopmental disorders, cognitive performance, educational performance) [55–58], particularly if prolonged and exclusive [59, 60]. The effect of breastfeeding is demonstrated in many observational studies and a cluster randomised controlled trial of assistance with breastfeeding [61]. Similarly, a meta-analysis of observational studies reports that children with autism are less likely to have been breastfed (OR = 0.61, 95% CI 0.45, 0.83) [57].

Causation is not easily established: infants may be exposed to medicines *in utero* and/ or via breastmilk, and observational studies cannot discount the possibility that difficulties with breastfeeding, and early discontinuation, are due to neonatal irritability associated with early signs of neurodevelopmental problems [62]. Also, some prescribed medicines (antidepressants, valproic acid derivatives) and high dose alcohol may simultaneously predispose to neonatal irritability [63], and disruption of breastfeeding physiology [48, 64, 65]. Accordingly:

- Breastfeeding warrants consideration as a health outcome measure, indicating a healthy mother-infant dyad.
- Those concerned with medicines’ safety in pregnancy and breastfeeding should account for infant feeding when modelling both long- and short-term outcomes.
- Inter-dependence between medicines and breastfeeding warrants scrutiny, alongside the full range of putative aetiologies of adverse outcomes. Currently, the optimum strategy is uncertain.

### How should breastfeeding be accommodated in pharmacoepidemiology?

Without information on breastfeeding, it will be impossible to separate the effect of exposure to medicines *in utero* and/or during breastfeeding from the effect of ‘not breastfeeding’ for some medicines. Although prescribed medicines and breastfeeding may affect infant development in different ways, information on breastfeeding is needed to understand and minimise adverse outcomes in childhood. With definitions (Table 1 Glossary), examples and explanation of implications, we explore breastfeeding in relation to confounding, mediating, moderating, and colliding [9], and offer diagrammatic illustrations as tentative directed acyclic graphs (DAGs) for discussion [66, 67]. Breastfeeding may be:

- A confounder, when exploring whether exposure both during and after pregnancy affects the infant, assuming medicines reach the infant *in utero* and then via breastmilk.
- A mediator, when exploring how *in utero* exposure affects neurodevelopment, when medicines may affect initiation or duration of breastfeeding.
- A moderator, by countering any adverse effects of medicines on neurodevelopment.
- Vulnerable to colliding and volunteer bias when exploring the effects of medicines on breastfeeding success.
- Incorporated into a range of statistical models.

### Confounding

#### Definition

The concept of ‘confounding’^glossary^, based on differences (or non-comparability) between exposed and non-exposed subjects, distinct from ‘selection bias’ ^glossary^, has developed in the last half-century [68]. The definition of confounder^glossary^ most widely adopted is: “a factor associated with both the exposure and the outcome, and not part of the causal pathway from exposure to outcome” [8], blurring effects [7, 69, 70] (Table 1).

All observational studies are vulnerable to confounding [67], including those considering the impact of maternal medicines on infant development. Some confounders are well known e.g., socioeconomic status (SES) ^glossary^; others are known, but accurate information is almost impossible to obtain in fieldwork and routine care e.g., doses of recreational drugs consumed. However, there remain ‘unknown unknowns’ or ‘lurking or latent variables^glossary^’ [71], such as local environmental pollution and cultural norms. Without randomisation, researchers can only speculate as to the effects of these unmeasured or unmeasurable confounders [13] on breastfeeding and childhood outcomes, based on knowledge of the data and their own *milltir sgwar* (communities, where they have lived for generations).

#### Example

**Fig. 1:**
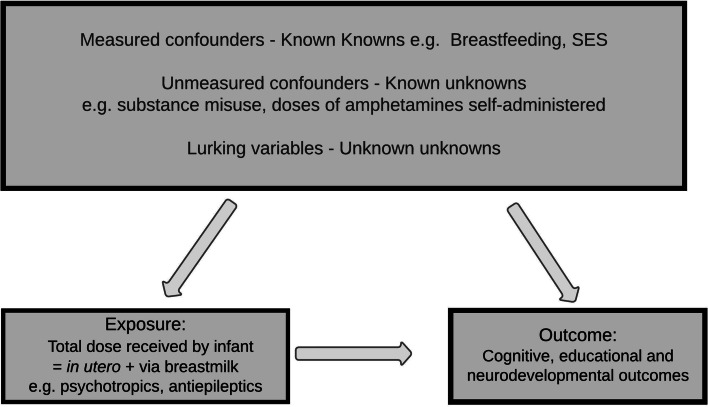
How does total medicine exposure affect infants’ neurodevelopment? Breastfeeding as a confounder. In this example, breastfeeding affects both neurodevelopmental outcomes and total exposure to medicines (via the placenta + breastmilk). This relates to postnatal exposure, following prenatal exposure

Breastfeeding would be considered a confounder in the analysis of the effect of medicines used both during and after pregnancy on neurodevelopmental outcomes when prescribed medicines do not greatly affect breastfeeding physiology and reach the infant *in utero* and via breastmilk. Most medicines enter breastmilk in small quantities [39]; therefore, breastfeeding affects both:

- neurodevelopment directly [57, 61], probably via biological processes and
- infants’ *total* medicine exposure during both prenatal and postnatal periods of brain development (Fig. 1).

#### Implications: infants’ total exposure, including via breastmilk

Identifying associations between infant outcomes and medicine exposure via breastmilk is complicated by variations in medicine transfer from breastmilk to infant, depending on dose, timing of administration and breastfeeding, and supplementary formula feeding. Concentrations of medicines in infant blood samples, as proportions of maternal blood concentrations, vary widely, for example: 28.9% (0.6–90.3%), 17.2% (12.4–22.0%), 21.4% (17.9–24.9%), and 44.2% (35.2–125.3%) (median and full range) for lamotrigine, topiramate, valproic acid, and zonasamide respectively [72]. Similarly, appreciable but variable and unpredictable concentrations of citalopram, sertraline, venlafaxine and metabolites pass into breastmilk [73]. This variation in infant exposure may relate to infants’ ability to metabolise and eliminate medicines. Elimination is compromised in premature or sick infants [39] or if maternal or infant metaboliser or transporter status is unusual [74], suggesting a need for close infant monitoring [75, 76]. Although prenatal antidepressant exposure is reported to adversely affect cognitive development [77–79], there are few data on long-term outcomes of exposure via breastmilk [65]. Short-term effects of exposure to psychotropic medicines via breastmilk include sedation, irritability, restlessness, diarrhoea and suboptimal weight gain [80, 81], but how these relate to long-term neurodevelopment is unknown. Current practice recommendations rest on case series, indicating that opioids, clozapine, amisulpride, combinations of central nervous system depressants, amiodarone, oral retinoids, radio-iodine, topical and systemic free iodine, and chemotherapy pass into breastmilk, risking (at least) short-term transgenerational (mother to child) adverse drug reactions (ADRs) ^glossary^ following breastmilk exposure [39, 82]. Including breastfeeding in analyses of infant outcomes informs families as to whether the benefits of breastfeeding outweigh the risks of increasing infants’ total dose. Analysis of IQ in children followed to age 6 suggests that there are no disbenefits of breastfeeding for children of mothers using sodium valproate (*n* = 35), but more data are needed regarding breastfeeding when prescribed phenytoin (*n* = 36) [83], particularly for rare ADRs, such as methaemoglobinaemia and combination regimens [65]. Larger studies are needed to resolve the dilemma “should those prescribed medicines breastfeed?” (see moderating, below).

### Mediating

#### Definition

**Fig. 2:**
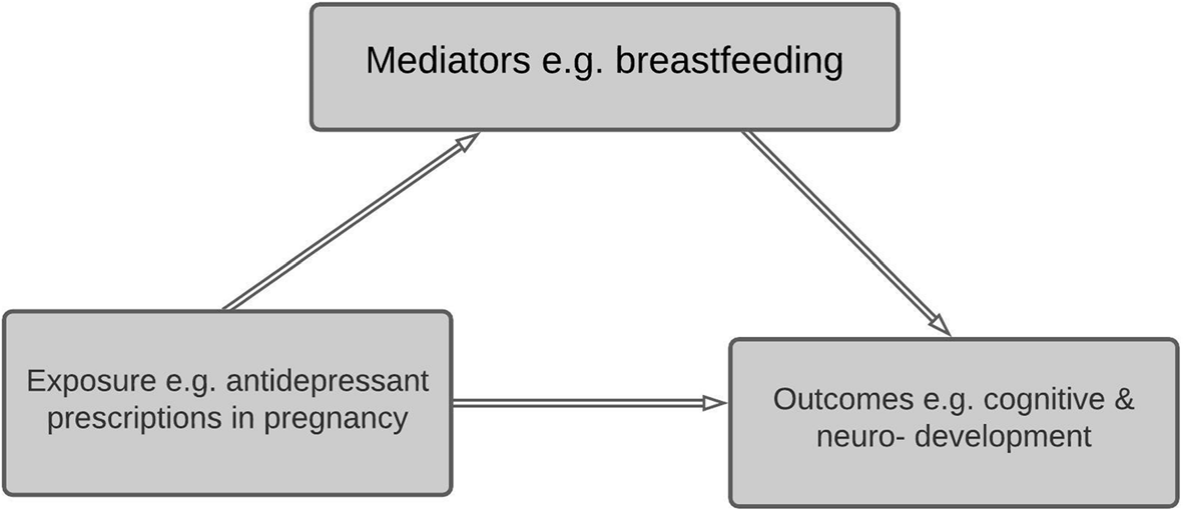
How do medicines in pregnancy affect infants’ neurodevelopment? Breastfeeding as a mediator. Breastfeeding as a mediator relates largely to prenatal exposure

Mediation occurs when the exposure is associated with an intermediate variable (the mediator), which is then associated with the outcome [84]. Mediators^glossary^, unlike confounders, lie on the causal pathway between exposure and outcome, and describe *how, or even why*, an association occurs [85, 86]. Breastfeeding is a mediator when:

- a) medicines reduce breastfeeding, and then.
- b) reduction or absence of breastfeeding affects neurodevelopment and health (Fig. 2).

Mediator effects can be tested by exploring the relationship between exposure and outcome with and without the mediator [13], which is particularly important for breastfeeding after *in utero* exposure [87].

#### example

SSRI antidepressant exposure in late pregnancy is associated with reduced breastfeeding rates [88] at birth [89], discharge [90], two [91], and 6–8 weeks [47], and, in some studies, with delayed neurodevelopment, including motor control [92], social behaviours [77], and autistic spectrum disorders [78, 79]. Neurodevelopmental delay following prenatal and perinatal antidepressant use may be due, in part, to the medicines’ effect on breastfeeding, i.e., delay may be mediated (or caused) by reduced breastfeeding caused by medicines.

#### Implications: infant development

Antidepressants *may* disrupt the physiology of lactation by delaying alveolar secretary activation from 69 to 86 hours, due to serotonin-dependent changes in tight (inter-cellular) junctions [48] and disruption of local production of serotonin [49, 64]. In addition to direct effects on lactogenesis, SSRI exposure in trimester 3 affects monoamine metabolism and serotonin availability in infants, associated with a dose–response increase in restlessness, tremor, and incoordination [93]. These symptoms, and any neonatal withdrawal symptoms of irritability, may impede latching, making breastfeeding painful and difficult, promoting discontinuation. The same disturbances that increase difficulties with breastfeeding may underlie delays in fine motor development at three years [92] or autistic-like behaviours [77]. Adult insomnia [94] and sleep disorders associated with SSRIs [95] and their effects on mother-infant bonding [96] and breastfeeding may compound any direct effects on lactogenesis.

If absence of breastfeeding contributes to any suboptimal neurodevelopmental outcomes associated with antidepressant exposure, breastfeeding partly mediates the association. Any mediator effects of breastfeeding on development can only be explored where a database collects data on children’s neurodevelopment *and* medicines *and* breastfeeding.

### Moderating

#### Definition

Moderators^glossary^ affect the strength or direction of the relation between exposure and outcome [13]. Moderators explain *when*, and under which circumstances, associations occur, and are sometimes used to identify subgroups at risk, e.g., age bands or co-morbidities, where exposure and outcome may be more closely linked than in the full population [84, 97]. Breastfeeding is a moderator if it affects:

- a) neurodevelopment directly via changes in neuronal architecture, *and*
- b) the extent of the impact of prenatal and postnatal medicine use on neurodevelopment.

In regression^glossary^ analyses, moderating effects are usually tested with interaction variables: if these explain a statistically significant amount of model variance^glossary^, moderator (or modification) effects are likely, i.e. associations identified depend on the value of the moderator [84], in this case, breastfeeding.

#### Example

If breastfeeding is a moderator, the impact of *in utero* exposure to medicines will depend on whether the infant is breastfed. In cohorts of infants exposed to antiepileptics (AEDs) (valproate, carbamazepine and lamotrigine) *in utero*, the prevalence of neurodevelopmental difficulties is lower in breastfed than formula-fed infants, despite the additional postnatal exposure [83, 98].

#### Implications: mitigating *in utero* exposure

**Fig. 3:**
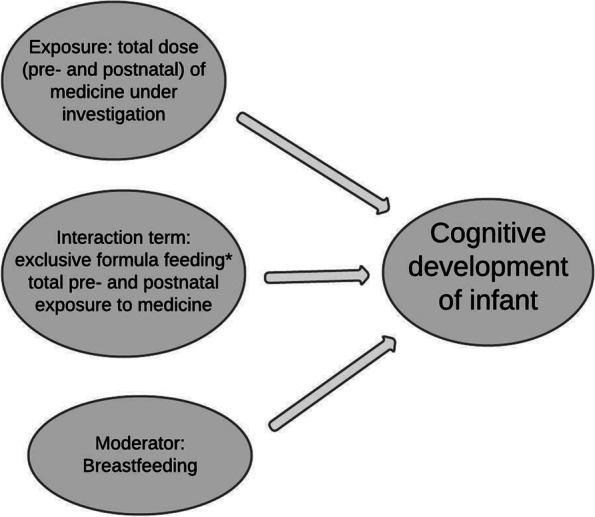
Does breastfeeding affect developmental outcomes for infants exposed to maternal medicines? Breastfeeding as a moderator. Breastfeeding may affect developmental outcomes if infants have been exposed to AEDs prenatally. This relates to prenatal exposure, followed by postnatal use of medicines

This suggests that breastfeeding *might* mitigate harm emanating from AEDs or other medicines, and exposures should be explored separately in breastfed and formula-fed infants (Fig. 3). This would inform families regarding the benefits of breastfeeding while using medicines.

Genetic variations might be considered as moderators, defining sub-groups at risk of exposure via breastmilk [72]. Transgenerational adverse outcomes may be confined to genotypes vulnerable to changes in serotoninergic and corticosteroid substrates [99]. Also, CNS depression or sedation in breastfed infants mainly occurs in infants with low activity in blood–brain barrier efflux transporters (P-gp) [74]. Allelic variations in transporter proteins [100], and maternal or infant single nucleotide polymorphisms [101] are rarely recorded at population level, but may define subgroups at risk of ADRs, and should be considered as moderators in *a priori* subgroup analyses.

### Colliding and Volunteer Bias: the case for whole population databases

Collider^glossary^ and volunteer bias^glossary^ are examples of selection bias^glossary^, defined as systematic differences between participants and non-participants (Table 1).

#### Definition

Collider bias is the distorted (induced) association between two or more variables that both affect the likelihood of an individual being included in the dataset (sampled) [102]. A collider is a variable influenced by other variables: for example, when an exposure or risk factor^glossary^ (such as medicines use) and an outcome (such as breastfeeding) both affect the likelihood of being sampled, they “collide”. Similarly, both being a ‘healthcare worker’ (exposure) and having a ‘severe COVID-19 infection’ (outcome) increase the chances of being tested for COVID, and thereby joining the dataset being analysed [102].

Volunteer samples may not represent the less affluent, smokers [21], or people with obesity [5]. *Selective or volunteer recruitment and any deficit in representativeness risks collider bias* [102]*.* This occurs when both exposure and outcome (or an antecedent of the outcome) influence recruitment or retention by their relation to volunteering, which then defines the sample [103–105]. The resulting collider bias can distort their relationships [102].

#### Example

**Fig. 4:**
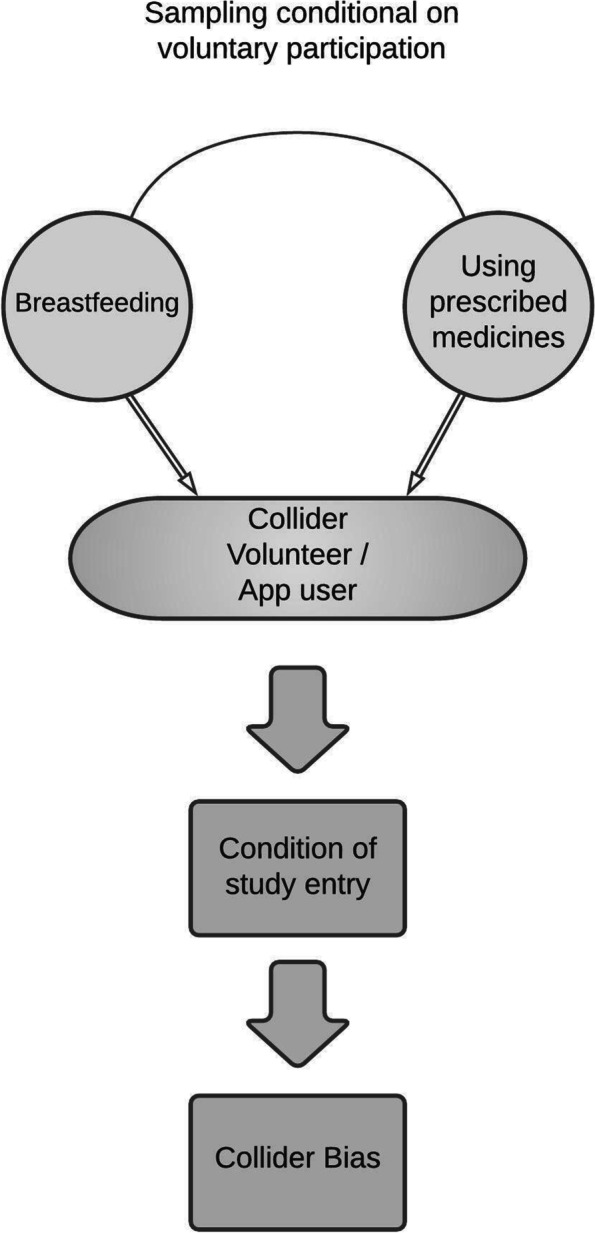
Does volunteer recruitment affect investigation of breastfeeding? Illustration of Collider Bias. In this example, the outcome is breastfeeding. Its relationship with maternal medicines is influenced by the composition of the sample of women studied. In this example, both breastfeeding and using prescription medicines affected recruitment to the study

Associations between variables may be vulnerable to collider bias [66, 106] if:

- Breastfeeding and medicine use both affect the selection of study participants, and
- the study sample over-represents these characteristics, (Fig. 4).

#### Implications: breastfeeding as a study outcome

When exploring the impact of medicines on initiation or duration of breastfeeding, if recruitment were to favour participants who a) were not using medicines, and b) breastfed, these characteristics would be over-represented. This over-representation would distort the sample and generate associations between breastfeeding and ‘no medicines’ that may not appear in the wider (non-volunteer) population [102]. An analogy might be drawn between volunteer bias and the “streetlight effect”: looking for lost items under a lamp-post, because that is the only place where anything can conveniently be seen [107]. Any associations found under the light may be due to colliding and co-existence in the illuminated patch: whilst they are valid in the sample examined, they may not be true in the wider population [102].

Capturing the whole population of a country or region removes volunteer bias, and hence collider bias, because neither exposure nor outcome nor covariates^glossary^ drive study inclusion. Attempts to account for colliding in the statistical analysis involve untestable assumptions, and it is better to avoid this problem by capturing data on the unselected regional or national population [102]. However, many databases are vulnerable to ‘live birth bias’, as they fail to record miscarriages [108], and infants dying within their first few days may not be linked to population databases [109], which means they do not capture all pregnancies, risking collider bias.

### Approaches to analysis

Regression^glossary^ models can identify associations, for example, between prescription medicines and exclusive formula feeding, but there are more complex questions, such as the inter-relationships between breastfeeding, maternal medicines, and infant development. Multilevel modelling^glossary^ extends regression analysis to account for clustering of individuals, for example by hospital, primary care provider, region, or country. This allows for the possibility that, in any one cluster, exposures may differ, for example, each primary care provider may have an individualised prescribing pattern. Further analytic techniques move beyond regression models to explore causation:

- **Marginal Structural Models**^glossary^ were designed to accommodate:
  - ◦ time-dependent exposure (e.g., one-off medicine administration, changes in treatment or drug absorption throughout pregnancy) and
  - ◦ time-varying covariates, such as breastfeeding, that may be both confounders (prenatal plus postnatal exposure) and mediators (prenatal exposure) (above) [110]

Where prescription regimens change frequently, analysis depends on modelling assumptions [111]. This approach has, for example, been used to explore associations between breastfeeding, SES and adult health [112], and between breastfeeding, infant deaths and self-reported ethnic group [113].

- **Structural Equation Modelling**.^glossary^ offers a framework for theory-driven hypotheses to be tested (or falsified) in a single cohesive model, using large datasets [14, 114, 115]. For example, this approach has been used to explore: the effects of maternal and infant characteristics on breastfeeding techniques and breastfeeding, [116] associations between attention-deficit-hyperactivity disorder (ADHD), obesity and breastfeeding [117], and predictors of infants’ neurodevelopment, including breastfeeding [115]

These’ causal models’ are used where trials would be considered unethical (pregnancy, breastfeeding) or impractical (rare outcomes), and are scarce [118]. However, models cannot accommodate the possibility that omitted variables^glossary^ may bias associations of interest [112]. In contrast, large trials account for known and unknown confounding variables by randomisation, albeit within the recruited population. Return on investment in analytic techniques will be insufficient without comprehensive data collection.

## Getting the full picture: challenges of comprehensive characterisation

Depending on how neurodevelopment is investigated, breastfeeding may be a confounder (when medicines are taken during pregnancy *and* whilst breastfeeding) or a mediator (when *in utero* exposure is considered) or a moderator (when defining ‘at risk’ subgroups) or a casualty of volunteer bias (in recruited cohorts). Many investigators consider breastfeeding sufficiently important to be an outcome itself [38], but it may be a casualty of volunteer bias in recruited cohorts [102].

Effective pharmacoepidemiology and pharmacovigilance need not only an understanding of causal pathways and unselected whole-population databases, but also comprehensive characterisation of the full range of variables affecting childhood outcomes [2, 119]. In addition to data on pregnancy dates, outcomes, and exposures to medicines and disease [2], regulators [1] recommend including data on: maternal age; obstetric and medical history; disease status and management; prescription of known teratogenic or foetotoxic medicines; folic acid and multivitamin use; smoking; alcohol intake; illicit drug use (with duration); lifestyle factors (exercise and nutrition); body mass index; and full family history of conditions possibly related to adverse perinatal and neurodevelopmental outcomes [2]. Breastfeeding is included as ‘follow up’ information [1]. Other parameters^glossary^ may be important in determining perinatal and childhood outcomes, for example: SES; parental educational outcomes; infections or inflammation in pregnancy; healthcare contacts (including, but not limited to, antenatal monitoring); vaccinations; rurality [120]; distance from environmental pollutants [121–123]; and genetic/ epigenetic influences [101].

**Environmental pollutants**, including lead, mercury, and dioxins, pass into breastmilk [124] and may increase the risk of sub-optimal growth [125], allergy [126] and neurodevelopmental delay [79, 127]; however, breastfeeding mitigates the impact of prenatal exposure [128]. Organochlorides may reduce lactation [129]. As with medicines, long-term effects of exposure via breastmilk are under-investigated, and databases contain little information.

Inter-relationships between these myriad variables determining childhood outcomes complicate evaluation of co-exposures, and may converge on SES^glossary^. SES is associated with: breastfeeding status [24, 38, 46]; environmental pollution [130]; health, perinatal, developmental and educational outcomes [131]; morbidity [132]; depression [133]; smoking; substance misuse; and prescription medicines [25, 47], including antidepressant prescribing [24, 38, 46, 134]. Combining these diverse variables into a propensity score risks overlooking individual modifiable risk factors and targets for change, such as prescribing practices or breastfeeding support. Subsuming the impact of ‘not breastfeeding’ under SES allows it to be ‘drowned out’, obscured, and lost to pharmacovigilance.

**Fig. 5:**
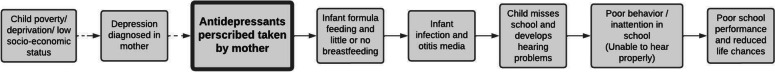
How is deprivation linked to school performance? A putative causal chain. School performance is affected by too many inter-related factors to be depicted in a single illustration. This figure illustrates just one scenario: we have prioritised clarity over complexity [103]

**Deprivation**^glossary^ [24, 38, 46], depression pre-pregnancy [47, 135] and antidepressants [47, 89–91] all lower breastfeeding rates [88, 136]. Both depression and antidepressants stimulate the hypothalamic–pituitary–adrenal axis, transfer of cortisol to the foetus, and epigenetic changes [137, 138] and their biological effects on neurodevelopment are difficult to disentangle [77]. Exactly how breastfeeding lies on the causal chain between deprivation (low SES) and poor school performance likely varies between individuals. Any impact of prescribed medicines on breastfeeding is of crucial importance, and any disruption of breastfeeding may have far-reaching consequences, as illustrated in Fig. 5.

The impact of prescribed medicines on reproductive health, childbirth and breastfeeding is not confined to transgenerational ADRs: other adverse effects, such as maternal weight gain, may affect breastfeeding directly or indirectly. For example, weight gain is associated with some antipsychotics, AEDs, antidepressants, and lithium; in turn, obesity complicates monitoring of pregnancy, glycaemic control, and childbirth [139, 140], and increases the risk of preterm birth, congenital anomalies and reduced breastfeeding [141–143].

## How did we get here? Controlling the databases

The Cumberlege Report states that pharmacovigilance systems failed and are failing pregnant individuals prescribed valproic acid derivatives, due to inability to monitor adverse outcomes [144]( p.4). Congenital anomalies following *in utero* valproate exposure were reported in 1982 [145] and 1985 [27], but were regarded as isolated cases or attributed to co-prescribing. After accumulation of twenty years’ data from large databases demonstrated an association between *in utero* sodium valproate exposure and lower IQ, [26] UK authorities issued unequivocal prescribing instructions, in 2018 [28]. A similar argument might be made to address the absence of breastfeeding data. In 1994 and 2001, the American Academy of Paediatrics [146] recommended codeine for short term cough suppression whilst breastfeeding [147]. Codeine had been noted to cause apnoea [148] or sedation [149] in breastfed infants a decade earlier, but was not contra-indicated whilst breastfeeding, until the death of a breastfed infant from codeine exposure was reported in 2005 [150]. This case is controversial [151], but other case series are reported [65], and several opioids are probably harmful *via* breastmilk [23, 81]. Only one large database study is available [152], and assessment of codeine exposure is complicated by its availability (in low doses) without prescription. Until databases include breastfeeding, risks remain that either some transgenerational ADRs will escape detection or decisions on medicines approvals will be based on case series, with inherent risks of mis-interpretation [151].

To monitor adverse effects, maternal prescription records should be linked to all childhood outcomes and modifiable risk factors [144], and analyses defined explicitly *a priori* [153]. The usefulness of databases depends on comprehensive coverage, and the nature and detail of their data, including ‘women’s problems’ of miscarriage, pregnancy termination, breastfeeding (extent and duration), and all infections (including those sexually transmitted). Database studies, unlike large clinical trials, cannot rely on randomisation to account for unrecorded variables. Omission or redaction of data constrains examination of potential associations and renders hypotheses unfalsifiable [154] (p.44). Hence, ‘facts’ uncovered by research are dependent on and limited by the processes of inquiry and the zeitgeist of investigators and those establishing and controlling the databases [155, 156]. Any discrimination in data collection may lead to omission of key variables, ‘blind spots’, and obfuscation of transgenerational ADRs. We can only speculate as to reasons for the apparently selective exclusions of crucial variables [144]. Without full transparency, these decisions may be heard as echoes of patriarchal power and its ability to control resources [157], with multi-generational consequences [144].

## Limitations of this paper

To our knowledge, this is the first paper to address the complex questions surrounding incorporation of infant feeding data into healthcare databases used to report patient safety. Other research approaches, such as case series, recruited cohorts and randomised trials, and analytical methods are detailed in guidelines [119], and are outside the scope of this discussion; however, concerns over external generalisation remain [21]. For simplicity, our tentative directed acyclic graphs do not offer comprehensive characterisation of the full range of variables affecting childhood outcomes and breastfeeding, listed above [1, 120, 122, 124, 129].

## Conclusions

Families need to know whether prescribed medicines will make breastfeeding more difficult and if breastfeeding will leave infants vulnerable to ADRs from maternal medicines or confer benefits, as in the wider population [55–61]. Current data are inconclusive [44, 158]. Existing regression analyses may offer sufficient evidence to target low-risk interventions to those in most need: for example, records of antidepressant prescriptions in pregnancy should trigger additional breastfeeding support [24, 88, 89]. However, answering complex questions on transgenerational ADRs and how to avoid, monitor and mitigate them, will involve juxtaposition of high-quality linked data on medicines, childhood outcomes and modifiable risk factors, including breastfeeding, in whole-population databases. Comprehensive characterisation and robust analyses of drug-related benefits and harms necessitate information across the life cycle, from miscarriage to fertility of the next generations: breastfeeding should not be a ‘blind spot’.

## Abbreviations

ADR: Adverse drug reaction
AED: Anti-epileptic drugs
DAG: Directed acyclic graph
MeSH: Medical Subject Headings (in PubMed)
OED: Oxford English Dictionary
PV: Pharmacovigilance
SES: Socioeconomic status
SSRI: Selective serotonin inhibitors (a class of antidepressants)
